# Supplementary material for: Strong negative nonlinear friction from induced two-phonon processes in vibrational systems
Source: Nat Commun. 2018 Aug 13;9:3241. doi: 10.1038/s41467-018-05246-w (PMC6089905; doi:10.1038/s41467-018-05246-w)
Supplement: Supplementary file 1 — Supplementary Information [file 41467_2018_5246_MOESM1_ESM.pdf]

Supplementary Information

Strong negative nonlinear friction from induced two-phonon processes in vibrational systems

Dong *et al*.

Supplementary Note 1. Dispersive coupling between the plate mode and the beam mode.

The plate mode and the beam mode are nonlinearly coupled. This parametric coupling arises from the tension induced in the beam as it deforms due to motion of one of the modes, which in turn modifies the spring constant of the other mode. Supplementary Figure 1a shows the linear dependence of the change in ω_1 on the square of the vibration amplitude a_2 of the beam mode. Supplementary Figure 1b shows a similar plot for the change in ω_2. The linear fits in Supplementary Figures 1a and 1b yield the parameters γ_{12} (9.80×10^{22} rad^2 s^{-2} m^{-2}) and γ_{21} (1.37×10^{25} rad^2 s^{-2} m^{-2}) respectively.

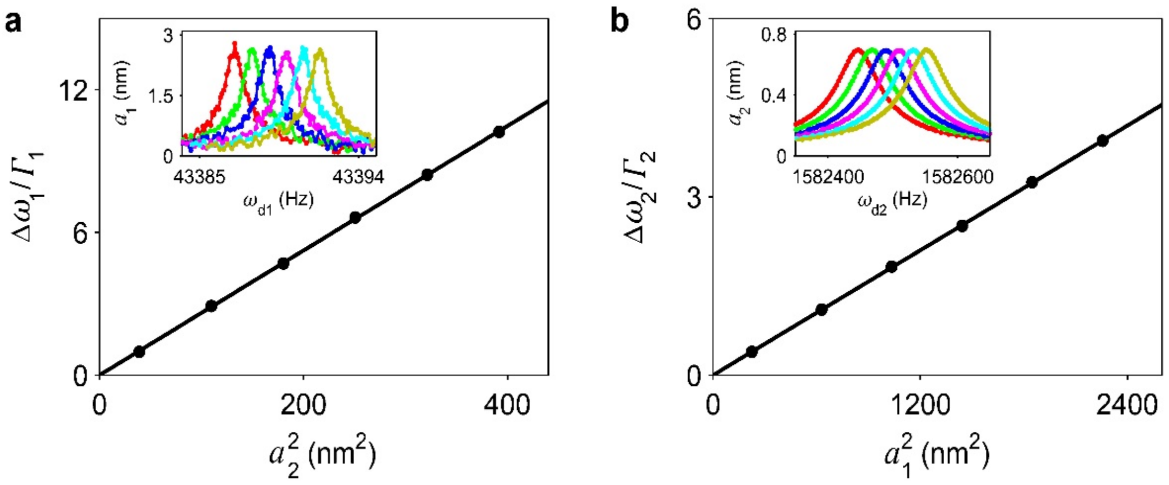

Supplementary Figure 1| Dispersive coupling between modes 1 and 2. (a) The dependence of the scaled shift of the resonance frequency of the plate mode $\Delta\omega_1/\Gamma_1$ on the vibration amplitude square of the beam mode a_2^2. The line represents a linear fit. Inset: spectra of the plate mode in response to a small probe voltage (Probe1 in Fig. 1a), with a_2^2 increased from 39.13 nm^2 in steps of 70.54 nm^2. **(b)** Same plot for the beam mode. In the inset, a_1^2 is increased from 223.93 nm^2 in steps of 406.47 nm^2.

Supplementary Note 2. Adiabatic approximation

The parameters of the effective Hamiltonian $\mathrm{H_{RWA}}$ are related to the parameters used in the equations of motion (1) and (2) as $\Lambda_{12} = \gamma C_{\mathrm{sc}}/2m_1 m_2 \omega_1 \omega_2$, $\Lambda_{ii} = 3\gamma_i C_{\mathrm{sc}}/4m_i^2 \omega_i^2$, $f_{\mathrm{p}} = F_{\mathrm{p}} C_{\mathrm{sc}}^{1/2}/4(2m_2\omega_2)^{1/2} m_1 \omega_1$. All these parameters have dimension of frequency.

The instantaneous decay rates for small vibration amplitudes (Figs. 2a and 2b) in the main text are calculated using Eq. (6) and the expression for α based on the adiabatic approximation with conservative nonlinear terms neglected. To extend the adiabatic approximation to larger amplitudes (Figs. 2c and 2d), it is necessary to re-introduce the conservative nonlinear terms. We define variables $\tilde{v}_{1,2}$ and ϕ as

$$v_1(t) = \tilde{v}_1(t) e^{\mathrm{i}\phi(t)} \quad (1)$$

$$v_2(t) = \tilde{v}_2(t) e^{-2\mathrm{i}\phi(t)} \quad (2)$$

The phase $\phi(t)$ will be chosen self-consistently so that, in the adiabatic approximation that we develop, the decay of function $\tilde{v}_1$ is not accompanied by phase accumulation, which means that $\tilde{v}_1$ can be set real.

From Eqs. (3) to (5) of the main text, equation for $\tilde{v}_2$ reads

$$\frac{\mathrm{d}}{\mathrm{d}t}\tilde{v}_2 = -D\tilde{v}_2 - \mathrm{i} f_{\mathrm{p}} \tilde{v}_1^{*2} \quad (3)$$

$$D = \Gamma_2 + \mathrm{i}\Delta - 2\mathrm{i}\dot{\phi} - \mathrm{i}\Lambda_{12}|\tilde{v}_1|^2 - \mathrm{i}\Lambda_{22}|\tilde{v}_2|^2 \quad (4)$$

Here, $D \equiv D(\dot{\phi}, |\tilde{v}_1|^2, |\tilde{v}_2|^2)$. The adiabatic approximation means that we set $\mathrm{d}\,\tilde{v}_2/\mathrm{d}t = 0$. We will then have to check that, for $\tilde{v}_2$ obtained this way, $|\mathrm{dlog}\,|\tilde{v}_2|/\mathrm{d}t| \ll |D|$. If $\mathrm{d}v_2/\mathrm{d}t = 0$, then

$$\tilde{v}_2 \approx -\mathrm{i} f_\mathrm{p} \tilde{v}_1^{*2} / D. \tag{5}$$

Substituting the result into Eqs. (3) to (5) of the main text with the account taken of Supplementary Equation 1, we find

$$\frac{\mathrm{d}}{\mathrm{d}t} \tilde{v}_1 = -\varGamma_\mathrm{ad}\, \tilde{v}_1 \tag{6}$$

$$\varGamma_\mathrm{ad} \equiv \varGamma_\mathrm{ad}(|\tilde{v}_1|^2) = \varGamma_1 - 2 f_\mathrm{p}^2 |\tilde{v}_1|^2 \,\mathrm{Re}\, D^{-1} \tag{7}$$

$$\dot{\phi} = \varLambda_{12} \left| f_\mathrm{p} \tilde{v}_1^{*2} / D \right|^2 + \varLambda_{11} |\tilde{v}_1|^2 - 2 f_\mathrm{p}^2 |\tilde{v}_1|^2 \mathrm{Im}\, D^{-1}\,. \tag{8}$$

Here $\dot{\phi}$ and parameter D are functions only of $|\tilde{v}_1|^2$. The numerical solution of the self-consistent nonlinear equations [Supplementary Equations (3) to (8)] gives the adiabatic decay rate $\varGamma_\mathrm{ad}$. From Supplementary Equation (5), this solution applies provided $\varGamma_\mathrm{ad} \ll |D|$, in which case $|\mathrm{d}\ \log \tilde{v}_2 / \mathrm{d}t| \ll |D|$, as it was assumed in the derivation.

Supplementary Note 3. Frequency and amplitude of self-sustained vibrations for negative friction

The frequency and amplitude of self-sustained vibrations can be derived from Eqs. (3) to (5) in the main text. By substituting $v_1 = c_1 e^{\mathrm{i}\delta\omega t}, v_2 = c_2 e^{-2\mathrm{i}\delta\omega t}$, we obtain

$$\dot{c}_1 = -(\varGamma_1 + \mathrm{i}\delta\omega) c_1 + \mathrm{i}\varLambda_{11} c_1 |c_1|^2 + \mathrm{i}\varLambda_{12} c_1 |c_2|^2 - 2\mathrm{i} f_\mathrm{p} c_1^* c_2^*, \tag{9}$$

$$\dot{c}_2 = -(\varGamma_2 + \mathrm{i}\varDelta - 2\mathrm{i}\delta\omega) c_2 + \mathrm{i}\varLambda_{12} c_2 |c_1|^2 + \mathrm{i}\varLambda_{22} c_2 |c_2|^2 - \mathrm{i} f_\mathrm{p} c_1^{*2} \tag{10}$$

For stationary self-sustained oscillations, $\dot{c}_1 = \dot{c}_2 = 0$. The relation between the amplitudes c_1 and c_2 and the value of $\delta\omega$ are given by equations

$$\Gamma_1|c_1|^2 = 2\Gamma_2|c_2|^2 \tag{11}$$

$$\delta\omega = (2\Gamma_1 + \Gamma_2)^{-1}\left[\Gamma_1\Delta - |c_1|^2\left(\frac{1}{2}\Gamma_1\Lambda_{12} + \frac{1}{2}(\Gamma_1^2/\Gamma_2)\Lambda_{22} - \Gamma_2\Lambda_{11}\right)\right] \tag{12}$$

where the squared amplitude of mode 1 is

$$|c_1|^2 = (\Gamma_2\,/\,\Gamma_1)G^{-2}\{\Gamma_1 G\,\Delta + (2\Gamma_1 + \Gamma_2)^2 f_{\mathrm{p}}^2 \pm (2\Gamma_1 + \Gamma_2)\left[2\Gamma_1 f_{\mathrm{p}}^2 G\Delta + (2\Gamma_1 + \Gamma_2)^2 f_{\mathrm{p}}^4 - \Gamma_1^2 G^2\right]^{1/2}\} \tag{13}$$

$$G = (\Gamma_1 + \Gamma_2)\Lambda_{12} + 2\Gamma_2\Lambda_{11} + \frac{1}{2}\Gamma_1\Lambda_{22} \tag{14}$$

The self-sustained oscillation frequency of the plate mode and the beam mode are $\omega_1 + \delta\omega$ and $\omega_{\mathrm{F}} - 2\omega_1 - 2\delta\omega$ respectively.

Supplementary Equations (11) to (14) describe two vibrational branches with different amplitudes and frequencies. Linearizing Supplementary Equations (9) and (10) about the corresponding solutions one can show that the larger-amplitude branch is stable, whereas the smaller-amplitude branch is unstable. The periodic solutions exist in the range where the argument of the square root in Supplementary Equations (13) and (14) for $|c_1|^2$ is positive, which imposes a constraint on the frequency and amplitude of the drive

$$2f_{\mathrm{p}}^2 G(\omega_{\mathrm{F}} - \omega_2 - 2\omega_1) + (2\Gamma_1 + \Gamma_2)^2 f_{\mathrm{p}}^4 - \Gamma_1^2 G^2 > 0 \tag{15}$$

This condition allowed us to calibrate the pumping power from the measured value of ω_{F} where the periodic solutions first emerge, because all other quantities in Supplementary Equation (15) are independently measured.

One can think of the birth of the stable and unstable limit cycles as a saddle-node bifurcation. There are limit cycles for the two modes, but since they are coupled, one can describe them near the bifurcation point by one variable, the effective radius of one of the cycles r . The equation for this radius near its bifurcational value r_0 is

$$\dot{r} = -(r - r_0)^2 + \epsilon \qquad (16)$$

where ϵ is the control parameter; in the experiment, the control parameter is the frequency detuning of the pump $\Delta = \omega_{\mathrm{F}} - \omega_2 - 2\omega_1$, and then $\epsilon \propto \Delta - \Delta_{\mathrm{B}}$ where Δ_B is the bifurcational value of Δ where the left-hand side of Supplementary Equation (15) is zero.

For $\epsilon < 0$ Supplementary Equation (16) has no stationary solutions for the limit cycle radius with r close to r_0. However, for $\epsilon > 0$ there are two stationary solutions, $r - r_0 = \pm\sqrt{\epsilon}$, which merge at $\epsilon = 0$. The solution with $r - r_0 = \sqrt{\epsilon}$ corresponds to a stable limit cycle, whereas the one with $r - r_0 = -\sqrt{\epsilon}$ corresponds to an unstable limit cycle. In the considered system, the trajectories that start inside the unstable limit cycle (with respect to the variables of the both coupled modes) go to the zero-amplitude state.

Supplementary Figure 2 shows the calculated amplitude of self-sustained vibrations as a function of pump detuning Δ. The range of Δ is expanded compared to the measurement and calculations shown in Fig. 3 of the main text. For the stable limit cycle (upper dark-colored curves), the amplitude increases with Δ. The unstable limit cycle (light-colored curves), on the other hand, shows a non-monotonic dependence of the amplitude on Δ. For large Δ, the amplitude of the unstable limit cycle increases with Δ. This behavior agrees with the notion that

it requires larger perturbations to excite stationary self-sustained vibrations as the pump frequency deviates more from the red-detuned secondary sideband.

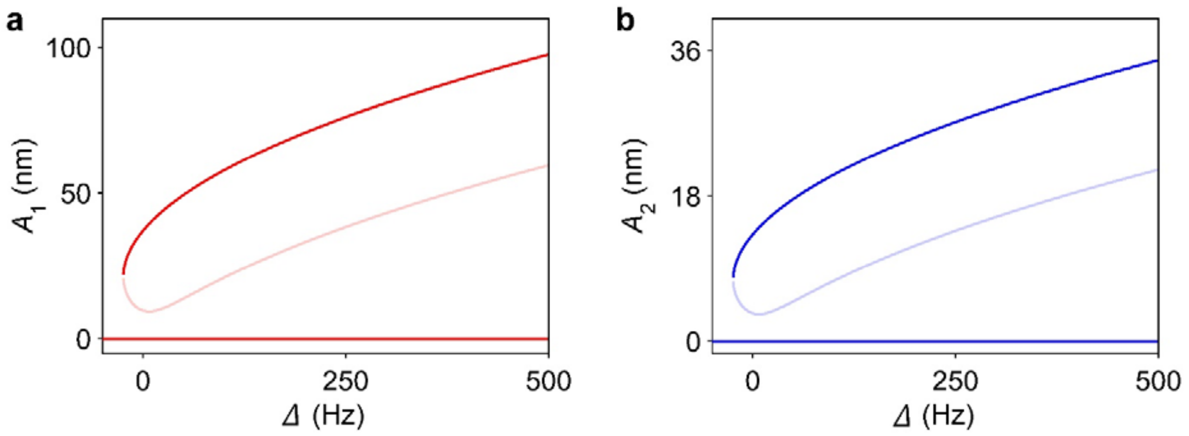

Supplementary Figure 2| Calculated amplitudes of self-sustained vibrations. (a) Amplitude of vibrations of mode 1 as a function of pump detuning Δ. The red curves represent the stable limit cycle and the zero-amplitude state. The light red curve corresponds to the unstable limit cycle. The results extend the theory curves in Fig. 3(a) of the main text, to a broader range of Δ. **(b)** The same plot for mode 2.

Supplementary Note 4. Multi-valued resonant response

Resonant response of the plate mode (mode 1) to a periodic force is described by adding a term $F_{\mathrm{d1}} \cos \omega_{\mathrm{d1}} t$ to Eq. (1), where F_{d1} is the driving amplitude and ω_{d1} is the resonant driving frequency with $|\omega_{\mathrm{d1}} - \omega_1| \ll \omega_1$. Equations (3) and (4) of the main text are modified to:

$$\dot{v}_1 = -\Gamma_1 v_1 + \mathrm{i}(\partial \mathrm{H}_{\mathrm{RWA}}/\partial v_1^*) - \mathrm{i} f_{\mathrm{d1}} \exp[\mathrm{i}(\omega_{\mathrm{d1}} - \omega_1)t] \quad (17)$$

$$\dot{v}_2 = -\Gamma_2 v_2 + \mathrm{i}(\partial \mathrm{H}_{\mathrm{RWA}}/\partial v_2^*) \quad (18)$$

where $f_{\mathrm{d1}} = (8 m_1 \omega_1 C_{\mathrm{sc}})^{-1/2} F_{\mathrm{d1}}$ is the scaled extra force ($f_{\mathrm{d1}} = 1.717\mathrm{s}^{-1}$ for fig. 4c of the main text).

To find periodic vibrations at frequency $\omega_{\rm d1}$ one solves Supplementary Equations (17) and (18) by setting $v_1 = u_1 \exp[\mathrm{i}(\omega_{\rm d1} - \omega_1)t]$, $v_2 = \mathrm{u}_2\exp[-2\mathrm{i}(\omega_{\rm d1} - \omega_1)t]$ and assuming that $u_{1,2}$ are independent of time, which reduces the problem to a set of algebraic equations for the complex amplitudes $u_{1,2}$

$$[\Gamma_1 + \mathrm{i}(\omega_{\rm d1} - \omega_1)]u_1 = \mathrm{i}\,\partial \mathrm{H}'_{\rm RWA}/\partial u_1^* - \mathrm{i}f_{\rm d1} \qquad (19)$$

$$[\Gamma_2 - 2\mathrm{i}(\omega_{\rm d1} - \omega_1)]u_2 = \mathrm{i}\,\partial \mathrm{H}'_{\rm RWA}/\partial u_2^* \qquad (20)$$

where $\mathrm{H}'_{\rm RWA}$ is given by Eq.(5) of the main text for $\mathrm{H}_{\rm RWA}$ in which v_1 is replaced by u_1 and v_2 is replaced by u_2 . The amplitude of forced vibrations is $a_1 = (2C_{\rm sc}/m_1\omega_1)^{1/2}|u_1|$. Supplementary Equations (19) and (20) can be further reduced to a system of two equations for the scaled squared vibration amplitudes

$$\left|Z_1 - 2f_{\rm p}^2|u_1^2|/\, Z_2^*\right|^2 |u_1^2| = |f_{\rm d1}|^2 \qquad (21)$$

$$|u_2|^2 = f_{\rm p}^2|u_1|^4 \,/\, |Z_2^2| \qquad (22)$$

$$Z_1 = \Gamma_1 + \mathrm{i}(\omega_{\rm d1} - \omega_1) - \mathrm{i}\Lambda_{12}|u_2^2| - \mathrm{i}\Lambda_{11}|u_1^2| \qquad (23)$$

$$Z_2 = \Gamma_2 - 2\mathrm{i}(\omega_{\rm d1} - \omega_1) + \mathrm{i}\Delta - \mathrm{i}\Lambda_{12}|u_1^2| - \mathrm{i}\Lambda_{22}|u_2^2| \qquad (24)$$

The phases of u_1, u_2 in the stationary states of forced vibrations can be immediately found from Supplementary Equations (19) to (24). The stability of the vibrational states is determined in the standard way by using Supplementary Equations (17) and (18) to write equations of motion for u_1, u_2 and linearize these equations about the stationary values of u_1, u_2.

The response curves for negative friction are qualitatively different from the response curves of the Duffing oscillator, as shown in Fig.4c of the main text. This figure shows that the

amplitude $|a_1|$ as function of the drive frequency can have disconnected branches in a certain range of the driving amplitude $F_{\mathrm{d}1}$. As explained in the main text, reaching a disconnected branch in this case requires activation, e.g., a pulse that makes the vibration amplitude sufficiently large so that it approaches the large-amplitude value. In other words, mode 1 must be brought by the pulse into the basin of attraction of the corresponding stable vibrational state. In Supplementary Figure 3 more detailed data on the response are shown and compared with the theoretical results described by Supplementary Equations (17) to (24). As seen from panels (a)-(c), at the end points of the large-amplitude branches the stable and unstable states merge. The analysis shows that these are simple saddle-node bifurcation points.

With increasing amplitude of the force $F_{\mathrm{d}1}$, the frequency range where there exists the isolated large-amplitude branch expands, Supplementary Figures 3a-c. At the same time, the amplitude of the vibrations on the small-amplitude branch increases. Ultimately, the two branches merge (Supplementary Figure 3d), and for still stronger drive (Supplementary Figures 3e and 3f) the response curve becomes reminiscent of the standard Duffing response curve for linear friction. The measurements and the theory are in good agreement.

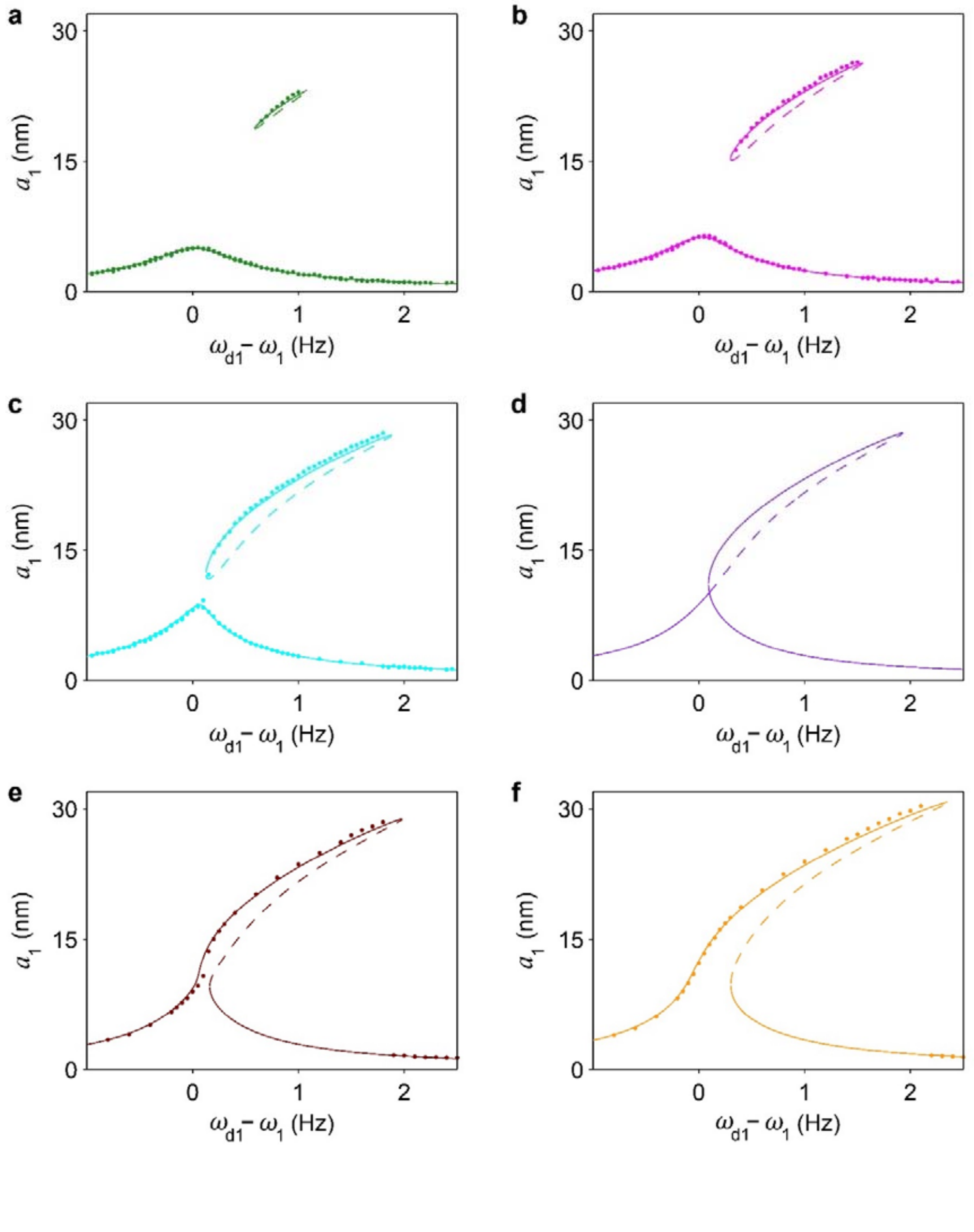

Supplementary Figure 3| Isolated branch in the response of forced vibrations. Measured (dots) and calculated stable (solid lines) and unstable (dashed lines) vibration amplitudes for the driving force amplitude F_{d1} equal to 0.595 pN **(a)** (green), 0.700 pN **(b)** (purple), 0.805 pN **(c)** (light blue), 0.8213525 pN **(d)** (dark purple) (calculation only), 0.840 pN **(e)** (brown) and 0.980 pN **(f)** (yellow). The isolated branch exists in a limited range of F_{d1}. As F_{d1} increases beyond a critical value, it merges with the lower branch [panel (d)] and disappears.

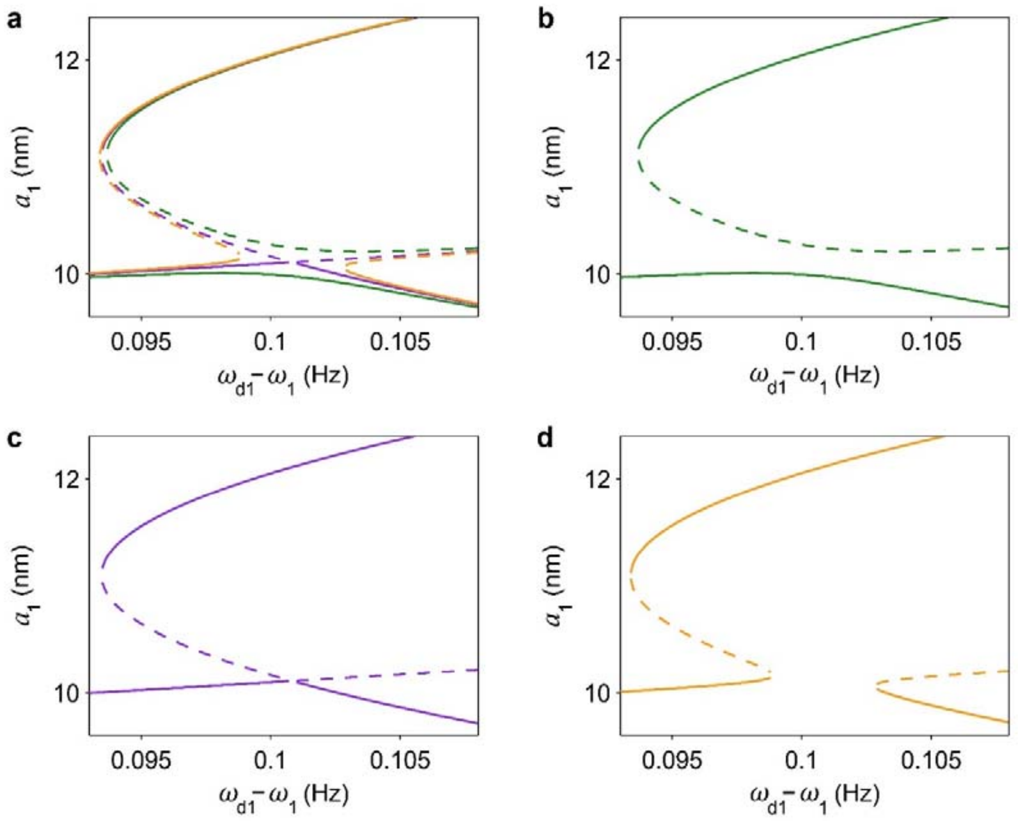

Supplementary Figure 4| Merging of the branches of forced vibrations near the bifurcation point. (a) Calculated stable (solid lines) and unstable (dashed lines) vibration amplitudes for F_{d1} equal to 0.8212748 pN (green), 0.8213525 pN (purple), and 0.8213798 pN (yellow) showing the merging of the isolated branch with the lower branch. Individual curves are plotted in **(b)**, **(c)** and **(d)**.

The merging and restructuring of the branches is an example of a bifurcation of co-dimension 2. Panel (a) in Supplementary Figure 4 shows the summary plot of the evolution of the frequency response curve with increasing drive, whereas panels (b)-(d) show the individual response curves; the drive amplitude in panel (d) is larger than in panel (b) by only 0.013%. The results are obtained by numerically solving equations for u_1, u_2. The overall evolution in the narrow range where the structure is changed can be mapped onto equation for an auxiliary variable x (a combination of u_1 and u_2) of the form $\dot{x} = x^2 - (\omega - \omega_c)^2 + \epsilon$, where ω_c is the value of $\omega_{d1} - \omega_1$ at the intersection of the curves in Supplementary Figure 4c and ϵ is the scaled

difference between F_{d1} and the value of F_{d1} in Supplementary Figure 4c. For $\epsilon > 0$, equation $\dot{x} = x^2 - (\omega - \omega_c)^2 + \epsilon$ has two disconnected (along the ω-axis) pairs of stationary solutions $x_{st} = \pm\sqrt{[\omega - \omega_c)^2 - \epsilon]}$. The frequency values $\omega_B = \omega_c \pm \sqrt{\epsilon}$ are saddle-node bifurcations. For $\epsilon = 0$ the saddle-node bifurcations merge, and for $\epsilon < 0$ there are only two branches of the stationary solutions in the considered range of ω.

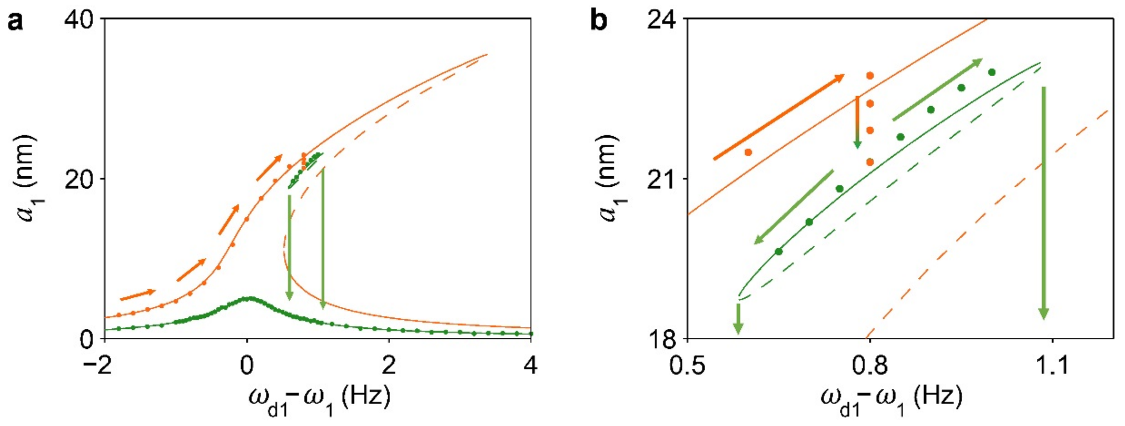

Supplementary Figure 5| Accessing the disconnected branch of forced vibrations. (a) Calculated stable (solid lines) and unstable (dashed lines) vibration amplitudes for F_{d1}= 0.595 pN (green) and 1.4 pN (orange). The calculated values are identical to Fig. 4d of the main text. Dots represent measurements. The orange arrows indicate how the isolated branch (green) can be accessed. The two green arrows show the amplitude jumps from the isolated branch to the lower branch at the two bifurcation points. **(b)** Close-up showing that the isolated branch can be reached by reducing F_{d1} from 1.4 pN to 0.595 pN in small steps with Δ fixed at 0.8 Hz.

Supplementary Figure 5 demonstrates one way to access the disconnected branch of stable vibrations (isolated branch in green). We first set the driving amplitude to be sufficiently large so that the frequency response resembles that of a Duffing oscillator (orange). The driving frequency is increased toward the bistable region, ensuring that the system resides in the high-amplitude vibration state. Upon reaching the target frequency, the driving amplitude is then gradually lowered so that the system settles into the isolated branch (Supplementary Figure 5b).

Once the system is on the isolated branch, if the driving frequency is increased (the green arrow that goes right in Supplementary Figure 5b) or decreased (the green arrow that goes left), at the corresponding bifurcation points the system switches to the low-amplitude branch.

Supplementary Note 5. Bistability of the characteristic spectral width $\Gamma_{\rm peak}$ for strong negative nonlinear friction

A sufficiently strong negative nonlinear friction gives rise to the isolated branch in the driven response of mode 1 as function of the drive frequency (Fig. 4c and Fig. 4d of the main text). The isolated branch and the lower branch have different maximum (as function of frequency $\omega_{\rm d1}$) amplitudes $a_{\rm 1max}$ of forced vibrations. Supplementary Figure 6 illustrates the effect of different strength of negative nonlinear friction on the quantity $\Gamma_{\rm peak}$ that is proportional to the ratio of the amplitude $F_{\rm d1}$ of the periodic driving force to $a_{\rm 1max}$. With large pump detuning Δ of -1000 Hz (black data), negative nonlinear friction is essentially absent and the frequency response has only one branch. It corresponds to the response of a Duffing oscillator with linear friction, and $\Gamma_{\rm peak}$ is independent of the drive amplitude $F_{\rm d1}$ even where the response to the resonant drive is significantly nonlinear[1]. When Δ is increased to -50 Hz (green data), the effects of negative nonlinear friction become important. As a consequence, $\Gamma_{\rm peak}$ decreases sharply at 1.12 pN, corresponding to a superlinear increase of the peak amplitude with drive amplitude. Upon further increase of Δ to -35 Hz and the respective increase of negative nonlinear friction, the response to the resonant drive displays two branches, as seen in Fig. 4c; see also Supplementary Note 4. We can define $\Gamma_{\rm peak}$ for each branch, and then it becomes multivalued for periodic driving amplitude between 0.57 pN and 0.82 pN (blue data). In particular, the two circles correspond to the maximum vibration amplitude for the two branches in Fig. 4c. Such

bistability of $\Gamma_{\rm peak}$ does not occur for systems with conservative nonlinearity such as Duffing or parametric resonators, nor in systems with weak nonlinear friction.

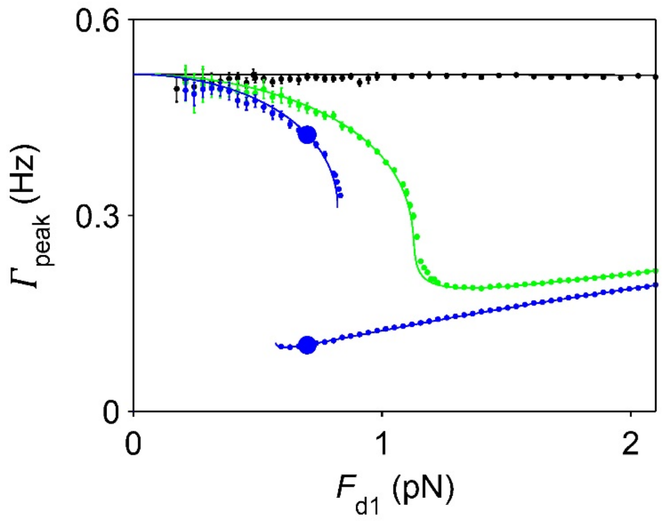

Supplementary Figure 6| Bistable behavior of $\Gamma_{\rm peak}$. The dots show the dependence of $\Gamma_{\rm peak} = F_{\rm d1}/2m_1\omega_1 a_{1\,\rm max}$ on the drive amplitude for negligible nonlinear friction (the pump detuning Δ = -1000 Hz, black) and increasingly strong negative nonlinear friction (Δ = -50 Hz, green, and Δ = -35 Hz, blue). The filled circles represent the peak amplitudes of the response $a_{1\rm max}$ for the two branches in Fig. 4c of the main text. The solid lines in this figure are theoretical predictions (Supplementary Note 4). Error bars represent ±1 s.e.

Supplementary Note 6. Hysteresis of the vibration amplitude with varying driving force amplitude.

The amplitude of forced vibrations of a nonlinear oscillator with linear friction is known to display hysteresis with varying amplitude of the driving resonant force[1]. Here we show that the character of the hysteresis changes qualitatively in the presence of negative nonlinear friction. In Supplementary Figure 7 we plot the amplitude of forced vibrations a_1 as a function of the driving force amplitude $F_{\rm d1}$ for a fixed driving frequency $\omega_{\rm d1}$. The data for red and purple

curves in Supplementary Figure 7 are taken with large pump detuning Δ = -1000 Hz, so that nonlinear friction effects are practically absent and the system behaves as a Duffing oscillator. For Duffing oscillators, the hysteretic response occurs only if the detuning $\delta\omega = \omega_{\mathrm{d1}} - \omega_1$ of the driving frequency from the mode eigenfrequency exceeds the threshold value of $\sqrt{3}\,\Gamma_1$ (~0.9 Hz for our system). Supplementary Figure 7 shows that when $\delta\omega$ is reduced from 1.1 Hz (blue curve) to 0.4 Hz (red curve), hysteresis indeed disappears. The red curve is obtained by changing the pump detuning to -35 Hz so that negative nonlinear friction becomes strong. The detuning of the driving force is set at $\delta\omega$ = 0.4 Hz. It is thus < $\sqrt{3}\,\Gamma_1$. Still we observe hysteresis, because vibrations at high amplitudes are stabilized by negative nonlinear friction. We note that the dependence of the vibration amplitude a_1 on the force amplitude F_{d1} for fixed ω_{d1} does not display an isolated branch, in contrast to the dependence of a_1 on ω_{d1} for fixed F_{d1}.

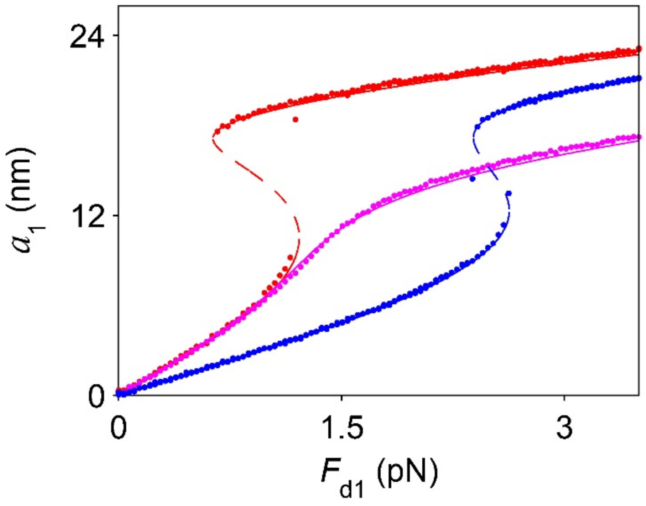

Supplementary Figure 7| The amplitude of forced vibrations with and without negative nonlinear friction. For a Duffing resonator with effectively linear friction (the pump detuning Δ = -1000 Hz), hysteresis is seen on the blue curve, which refers to $\omega_{\mathrm{d1}} - \omega_1$ = 1.1 Hz > $\sqrt{3}\,\Gamma_1$. For the purple curve with $\omega_{\mathrm{d1}} - \omega_1$ = 0.4 Hz smaller than $\sqrt{3}\,\Gamma_1$, there is no hysteresis. In contrast, when negative nonlinear friction is strong (Δ = -35 Hz), hysteresis is seen

even for $\omega_{d1} - \omega_1$ = 0.4 Hz (the red curve). Solid and dashed lines are calculated stable and unstable vibration amplitudes respectively. Dots represent measurement.

Reference

1. Landau L. & Lifshitz E. *Mechanics* (Elsevier, Amsterdam, 2004).
